# Adsorption and Thermal Evolution of the Carbonyl‐functionalized Ionic Liquid [5‐oxo‐C_6_C_1_Im][NTf_2_] on Pt(111): A Combined IRAS, STM, and DFT Study

**DOI:** 10.1002/chem.202403900

**Published:** 2024-12-10

**Authors:** Hanna Bühlmeyer, Lukas Knörr, Julien Steffen, Roman Eschenbacher, Jonas Hauner, Andreas Görling, Jörg Libuda

**Affiliations:** ^1^ Interface Research and Catalysis ECRC Friedrich-Alexander-Universität Erlangen-Nürnberg Egerlandstraße 3 91058 Erlangen Germany; ^2^ Chair of Theoretical Chemistry Friedrich-Alexander-Universität Erlangen-Nürnberg Egerlandstraße 3 91058 Erlangen Germany

**Keywords:** Ionic liquids, Infrared reflection absorption spectroscopy, Scanning tunneling microscopy, Pt(111), SCILL

## Abstract

The coating of heterogeneous catalysts with ionic liquids enables precise tuning of catalytic activity and selectivity. Recently, the fundamentals of solid catalysts with ionic liquid layers have been extensively studied. So far, investigations have focused on simple ILs without specialized functional groups. In our current work, we aim to involve functionalized ILs to take advantage of the interactions between these functional groups, the catalyst, and the reactants. In this study, we investigated the interaction, and thermal stability of the carbonyl‐functionalized IL [5‐oxo‐C_6_C_1_Im][NTf_2_] on Pt(111) by infrared reflection absorption spectroscopy and scanning tunneling microscopy. In addition, we performed density functional theory calculations to support our interpretation. At 200 K and low coverage, the carbonyl group of the [5‐oxo‐C_6_C_1_Im]^+^ cation is oriented parallel to the Pt(111) surface. With increasing coverage, the alkyl chain detaches from the surface and orients towards the vacuum. The [NTf_2_]^−^ anion adsorbs parallel to the surface via the oxygen atoms of the SO_2_ groups. At higher coverage, at least one of the SO_2_ groups completely detaches from the surface. Upon heating to 250 K, we observe decomposition and partial desorption of [5‐oxo‐C_6_C_1_Im][NTf_2_], with further decomposition and desorption occurring between 350 and 400 K.

## Graphical abstract

In solid catalysts with ionic liquid layers (SCILL), ionic liquid (IL) coatings are used to modify noble mtal catalysts. The functionalised IL improves the SCILL by increasing the selectivity, with the functional group playing a crucial role in this process. To understand the mechanisms behind this selectivity control, we performed model studies using surface science techniques under ultra‐high vacuum conditions.

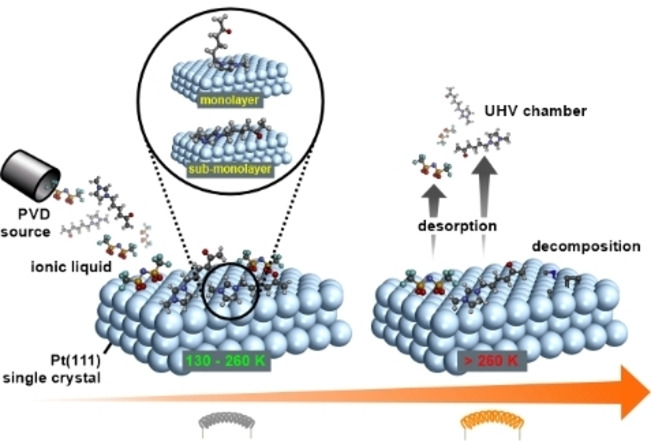

## Introduction

Advanced catalytic materials are essential to improve sustainability of chemical processes. In this respect, the selectivity of the catalyst is key.^[1]^ A high selectivity results in a high amount of the desired product while minimizing side products.^[2]^ A promising concept for improving the selectivity is the solid catalyst with ionic liquid layer (SCILL).[^3^, ^4^, ^5^, ^6^, ^7^, ^8^, ^9^, ^10^, ^11^, ^12^, ^13^] In this approach, conventional noble metal catalysts are modified by a thin film of ionic liquid (IL). It is noteworthy that, in spite of the novelty of the approach, SCILL systems for the selective hydrogenation of acetylene are already used at industrial scale.[^7^, ^14^]

The improved selectivity can originate from different effects: Firstly, there is the interaction of the IL with the catalyst surface. For example, specific active sites of the catalyst surface can be blocked for the reactant by ligand effects.[^3^, ^5^, ^8^, ^15^, ^16^, ^17^, ^18^] In addition, decomposition of different ILs has already been observed on reactive surfaces such as Cu,^[19]^ Ag,^[20]^ and Pt.[^21^, ^22^] These decomposition products can also change the catalytic properties of the catalyst. Secondly, the IL can interact with the reactant itself. The different solubilities of the individual reactants in the IL can influence the concentration of the reactants at the active sites.[^6^, ^17^, ^23^] Another effect to be taken into account are lateral interactions between different coadsorbates on the surface. The activation barrier at an interface is determined not only by the interaction of the reactant with the surface, but also by intermolecular interactions with coadsorbates. This means that the coadsorbates have a significant effect on the activation energy and, thus, on the selectivity for a particular reaction pathway.

One specific example of a selective hydrogenation reaction of great interest is the heterogeneous catalyzed hydrogenation of carbonyl compounds.[^24^, ^25^] Activation of the stable C=O bond for the hydrogenation reaction is the key step in these reactions.^[24]^ It has been demonstrated that this can be achieved by keto‐enol tautomerization. Hydrogenation of the C=C group of the enol species has a lower activation‐barrier than hydrogenation of the C=O group of the keto species.[^26^, ^27^, ^28^] However, the enol alone is not stable and readily converts back to the keto form.^[29]^ Recent studies on a carbonyl compound, namely acetophenone, showed that ketone‐enol dimers are formed on Pt(111) under certain reaction conditions.[^30^, ^31^, ^32^] Here, the typically unstable enol is stabilized by lateral interactions via hydrogen bonds with the ketone.

The chemical versatility of the ILs[^9^, ^33^] allows us to tailor SCILL systems for very specific reactions, where the IL can be used as a coadsorbate and interact with the reactants via a functionalized group. As a starting point, we study the behavior of the functionalized IL with the metal surface itself. In this work, we investigate the adsorption, interaction, and thermal stability of the carbonyl functionalized IL 1‐(5‐oxo‐hexyl)‐3‐methyl‐imidazolium‐bis‐(trifluormethylsulfonyl)‐imid [5‐oxo‐C_6_C_1_Im][NTf_2_] on Pt(111). To this aim, we combine infrared reflection absorption spectroscopy (IRAS) in time‐resolved and temperature‐programmed experiments under ultrahigh vacuum (UHV) conditions with scanning tunneling microscopy (STM) measurements and density functional theory (DFT).

## Experimental

**IRAS**. The in‐situ sample preparation and IRAS experiments were conducted in an ultra‐high vacuum (UHV) setup with a base pressure of 1.0×10^−10^ mbar. The setup is divided in two sub‐chambers. The first chamber was used for sample cleaning and included an ion gun (Specs IQE 11/35), a quartz crystal microbalance (QCM, Inficon SQM‐160), a LEED optics (Specs ErLEED 150), a gas dosing system and a quadrupole mass spectrometer (QMS, Blazers Quadstar 422). The second sub‐chamber included a remote‐controlled effusive beam source, several custom‐built Knudsen cells, and a QMS (Hiden Analytical Hal 3 F). A vacuum Fourier‐transform infrared (FTIR) spectrometer (Bruker VERTEX 80v) equipped with a liquid nitrogen cooled mercury cadmium telluride (MCT) detector was used for the IR spectra.

The Pt(111) single crystal (MaTeck) was cleaned by multiple cycles of Ar^+^ sputtering (Ar, Linde, >99.9999 %; 1.6 keV, 8.5×10^−5^ mbar, 30 min). Afterwards, the sample was annealed at 1150 K for 10 min in UHV as well as in O_2_ atmosphere (1.0×10^−6^, Linde, purity 99.999 %). This was followed by a heating step to 1175 K under UHV to remove residual O_2_. The cleanliness of the sample was checked by IRAS via adsorption of CO at 300 K (1×10^−6^ mbar, Westfalen 99.97 %). Traces of carbonyls were removed from the supplied CO via a home‐built liquid nitrogen trap. After stepwise CO adsorption, the sample was cleaned again by an additional sputtering and annealing cycle.

[5‐oxo‐C_6_C_1_Im][NTf_2_] was evaporated from a glass reservoir using a home‐built Knudsen cell. The evaporator was isolated from the main chamber by a gate valve, which was connected to a separate high vacuum line. The evaporator was heated for 20 minutes with the gate valve closed before the experiments. The deposition was monitored by simultaneous acquisition of IR spectra (1 min/spectrum, spectral resolution of 4 cm^−1^). Each spectrum was then referenced to the spectrum of the clean surface recorded before deposition.

For the TP‐IRAS experiments, the temperature of the sample was ramped with a rate of 2 K/min, while simultaneously recording IRA spectra (1 min/spectrum). The attenuation of the IR signals with increasing temperature was corrected for by normalization of the spectra as previously described by Xu et al.^[34]^

**STM**. The sample preparation and STM measurements were performed in a SPECS UHV system with a base pressure <1.0×10^−10^ mbar consisting of two sub‐chambers. The sample was cleaned and prepared in the preparation chamber. The STM measurements were carried out in the analysis chamber with an SPM 150 Aarhus microscope. In previous publications, a detailed description of the setup can be found.[^22^, ^35^, ^36^]

The Pt(111) single crystal (MaTeck) was cleaned by Ar^+^ sputtering (Ar, Linde, >99.9999 %; 1.0 keV, 3×10^−5^ mbar, 45 min), and subsequent annealing (1150 K in UHV; 12 min). The cleanliness of the single crystal was checked with STM.

The IL was evaporated from a home‐built Knudsen cell in form of a glass reservoir loaded with [5‐oxo‐C_6_C_1_Im][NTf_2_] on a cooled Pt(111) sample. The evaporator is separated from the main chamber by a gate valve and pumped via a separate high vacuum line. For deposition, the evaporator was preheated for 60 minutes. After preparation, the sample was quickly transferred to the analysis chamber. For low temperature measurements, the STM was cooled with liquid nitrogen while the scanner unit was counter‐heated to RT. For temperature‐dependent experiments, the STM was heated together with the sample to the desired temperature and kept at this temperature for several hours. Afterwards, the STM and the sample were cooled back down to the measuring temperature.

The STM images were recorded in constant current mode with a tungsten SPECS Kolibri sensor, using a bias voltage of∼1.0–1.6 V applied to the sample and a tunneling current of∼ 100–350 pA. The images were treated and evaluated with Gwyddion software. More detailed preparation and scanning parameter can be found in Table S1.

**DFT**. Periodic DFT modeling of the system was carried out with the VASP code, in which a plane wave basis set for the description of the valence electrons is used in combination with the projector augmented wave (PAW) method for the representation of the atomic cores.[^37^, ^38^, ^39^]

The kinetic energy cutoff was chosen to be 500 eV for metal surface slab and adsorbate optimizations, exchange correlation effects were treated with the PBE functional.^[40]^ The DFT D3 correction was used for the better description of dispersion interactions in the case of adsorbate optimizations and also in the case of clean surfaces to ensure a common energy scale.[^41^, ^42^]

The Pt(111) surface was modeled as surface slab being five atom layers deep, where the lowest two layers were held fixed during molecular dynamics trajectories and geometry relaxations. For the [5‐oxo‐C_6_C_1_Im][NTf_2_] pairs, surface slabs of different sizes (3×3, 4×4, 5×5, 6×6, and 7×7) were used. The dimensions of the surface slabs were obtained by first optimizing the volume of fcc Pt bulk. The optimized Pt−Pt distance of 2.804 Å was used for the Pt(111) surface slabs, the non‐fixed layers were optimized subsequently. A vacuum separation of 25 Å between the periodic images of the Pt surface was introduced in order to ensure enough space between them also in the case of added adsorbates and desorbed adsorbates between the layers. Brillouin zone sampling was done with a Γ‐containing 3×3×1 k‐point mesh for the IL pairs in the 7×7 and 6×6 unit cells, a Γ‐containing 4×4×1 k‐point mesh for those in the 5×5 unit cell and a Γ‐containing 5×5×1 k‐point mesh for those in the 4×4 and 3×3 unit cells. A Methfessel‐Paxton scheme 1^st^ order with a broadening of 0.15 eV was applied to smear the electronic states.^[43]^ To optimize the adsorption energies, the IL molecules were optimized separately in a 25×25×25 Å box, applying a Gaussian smearing with a broadening of 0.04 eV.

The geometries of the model surfaces and the gas phase IL molecules were relaxed until all gradient components were below 0.01 eV/Å. The IL molecules were placed on the surface with the build adsorbates.py script from the VASP4CLINT repository [https://github.com/Trebonius91/VASP4CLINT]. For the flexible IL pairs, three different initial positions were built per unit cell size. Following this, they were annealed with a short NVT dynamics trajectory (Nose‐Hoover thermostat,[^44^, ^45^] 700 K, 1 fs time step). Each trajectory was calculated for roughly one week on 576 CPUS, leading to average trajectory lengths of 1600 steps (7×7), 3300 steps (6×6), 7500 steps (5×5), 17900 steps (4×4) and 51200 steps (3×3). The time‐dependent potential energy was analyzed and the frame with the lowest potential energy was chosen to be the initial structure of the subsequent geometry optimization. All structures were then optimized until all gradient components were below 0.02 eV/Å. For the calculation of adsorption energies, the clean metal surfaces as well as the molecules in a cubic box were optimized separately and their energies calculated.

Numerical frequencies and IR spectra of the adsorbates were calculated by elongating the atoms of the adsorbates and holding the Pt surface atoms fixed. The metal surface selection rule is effective for the IR intensities since the dipol correction in the z‐direction (IDIPOL=3) has been activated. The split freq program from the VASP4CLINT github repository has been used to manage the calculations of the larger adsorbate systems and to obtain the IR spectrum.

## Results and Discussion

### Adsorption of [5‐oxo‐C_6_C_1_Im][NTf_2_]

**Figure 1:**
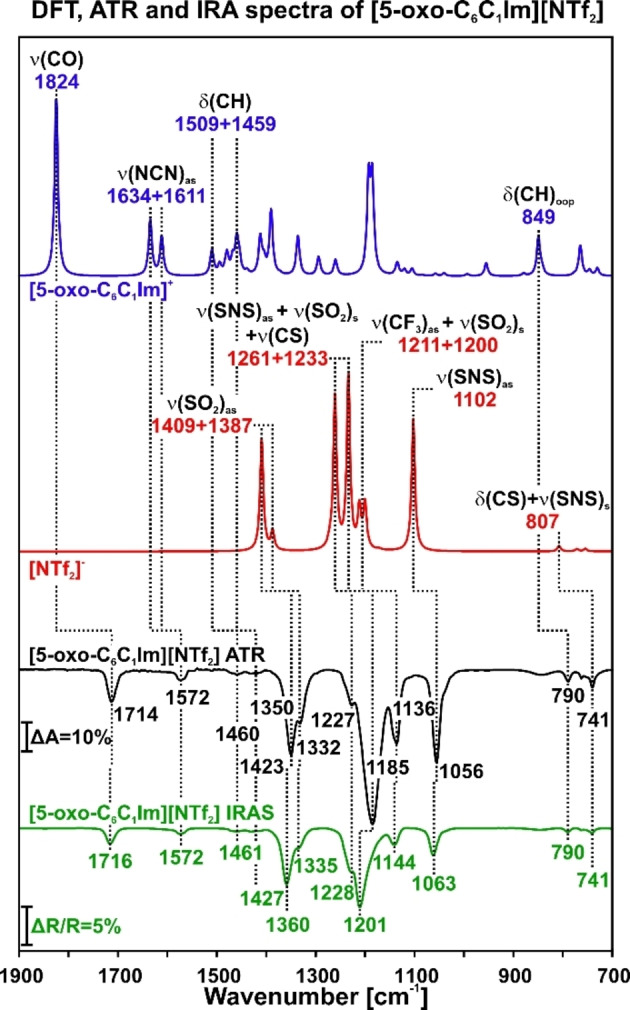
Comparison of the DFT, ATR IR, and IRAS spectra of [5‐oxo‐C_6_C_1_Im][NTf_2_]. The DFT spectra shows the isolated cation and anion. The ATR IR spectrum represents the IL of the bulk, and the IRAS data is obtained from a multilayer of the IL on Pt(111) at 300 K.

**Multilayer**. In order to determine whether [5‐oxo‐C_6_C_1_Im][NTf_2_] evaporates without decomposition and to assign the IR bands, we compared the IRAS spectra of a multilayer film deposited by physical vapor deposition (PVD) with ATR IR measurements and DFT calculations. The IL was deposited onto a clean Pt(111) surface at 300 K until a multilayer was formed. In Figure 1, we present the DFT spectra for the cation [5‐oxo‐C_6_C_1_Im]^+^ and the anion [NTf₂]^−^, in conjunction with the ATR IR spectra and IRAS spectra of the IL multilayer film. For the [5‐oxo‐C_6_C_1_Im]^+^ cation, the DFT data shows a band at 1824 cm^−1^ corresponding to the ν(CO) vibration. The bands at 1634 and 1611 cm^−1^ are assigned to the ν(NCN)_as_ vibrations, while the bands at 1509 and 1459 cm^−1^ correspond to the δ(CH) vibrations. We assign the band at 849 cm^−1^ to the δ(CH)_oop_ vibrations. For the [NTf₂]^−^ anion, the DFT data indicates that the bands at 1409 and 1387 cm^−1^ contribute to the ν(SO₂)_as_ vibration. We assign the bands at 1261 and 1233 cm^−1^ to a combination of ν(SNS)_as_, ν(SO₂)_s_, and ν(CS) vibration modes. The bands at 1211 and 1200 cm^−1^ both contribute to the ν(CF₃)_as_ vibration mode, while we assign the band at 1102 cm^−1^ to the ν(SNS)_as_ vibration mode. We attribute the band at 807 cm^−1^ to δ(CS) and ν(SNS)_s_ vibrations.

In Table 1, we summarize the assignment of the DFT, ATR IR, and IRAS bands. These assignments are based on DFT calculations and previous IRAS studies of similar ILs.[^46^, ^47^] The ATR and IRAS spectra show similar shapes, with only minor differences in band positions. However, the IR bands calculated via DFT differ from the experimental values due to molecular interactions, molecule‐interface interactions, and packing effects. The excellent agreement of the ATR IR and IRAS results indicate that the IL was evaporated under UHV conditions without decomposition.

**Table 1:** Assignment of the DFT, ATR, and IRA spectra of [5‐oxo‐C6 C1Im][NTf2].

DFT [cm^−1^]	ATR IR [cm^−1^]	IRAS [cm^−1^]	Assignment
1824	1714	1716	ν(CO)
1634+1611	1572	1572	ν(NCN)_as_
1509+1459	1460+1423	1461+1427	δ(CH)
1409+1387	1350+1332	1360+1335	ν(SO_2_)_as_
1261+1233	1227+1185	1228+1201	ν(SNS)_as_+ν(SO_2_)_s_+ν(CS)
1211 + 1200	1136	1144	ν(CF_3_)_as_
1102	1056	1063	ν(SNS)_as_
849	790	790	δ(CH)_oop_
807	741	741	δ(CS)+ν(SNS)_s_

Furthermore, we analyzed the growth of a multilayer of [5‐oxo‐C_6_C_1_Im][NTf_2_] on Pt(111) at 130 K. The IL was deposited via PVD while IRAS spectra were recorded every minute, with the sample maintained at a constant temperature of 130 K (see **Supporting Information**). In Figure S2, we present the spectra recorded during the growth of the film, along with the band assignments. The bands are at identical positions as in the previous experiment and can be assigned accordingly (see Figure 1 and Table 1).

**Figure 2:**
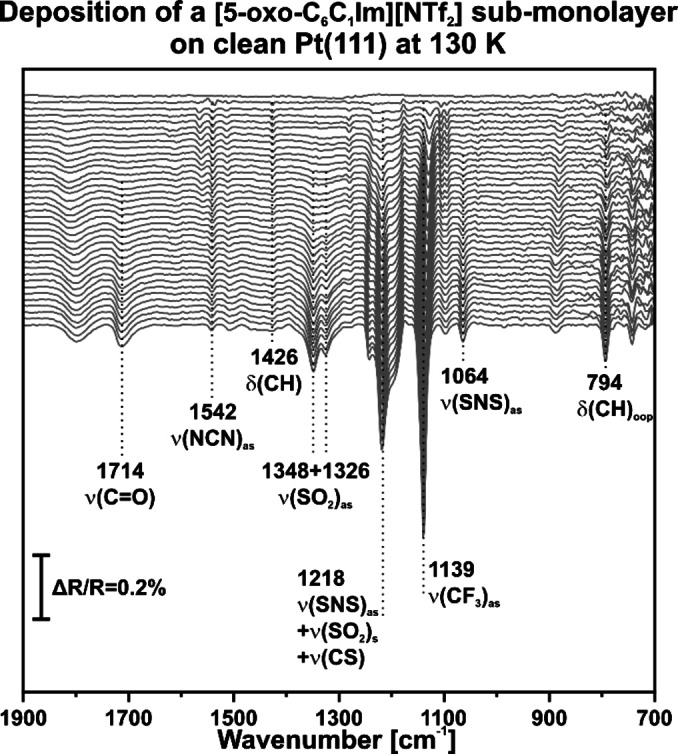
Deposition of a [5‐oxo‐C_6_C_1_Im][NTf_2_] sub‐monolayer on a clean Pt(111) surface at 130 K recorded by time‐resolved IRAS.

**(Sub−)monolayer**. To better understand the interaction between [5‐oxo‐C_6_C_1_Im][NTf_2_] and the Pt(111) surface, we performed a sub‐monolayer experiment. We deposited a sub‐monolayer by PVD at 130 K and continuously recorded in situ IRAS spectra at one‐minute intervals during the deposition (Figure 2).

The initial spectrum refers to the clean Pt(111) surface without IL. Upon IL deposition, two bands appear at 1542 and 1426 cm^−1^, corresponding to the ν(NCN)_as_ and δ(CH) vibrations of the cation, respectively. With increasing coverage, new bands emerge at 1218, 1139, and 794 cm^−1^. We assign the band at 1218 cm^−1^ to the combination vibration of ν(SNS)_as_, ν(SO₂)_s_, and ν(CS). The bands at 1139 and 794 cm^−1^ are attributed to the ν(CF₃)_as_ and δ(CH)_oop_ vibrational modes, respectively.

At even higher coverage, additional bands at 1714, 1348, 1326, and 1064 cm^−1^ appear. The first band corresponds to the ν(CO) vibration of the cation, while the others are assigned to the ν(SO₂)_as_ and ν(SNS)_as_ modes of the anion. We attribute the band at 1811 cm^−1^ to the ν(CO) vibration of carbon monoxide adsorbed in a bridge position,^[48]^ indicating surface contamination by carbon monoxide. Small contaminations by CO picked up from the background are inevitable under the present conditions.

Note that due to the metal surface selection rule (MSSR), only dynamic dipole moments perpendicular to the surface are detected by IRAS.^[49]^ Therefore, the delayed appearance of the ν(CO) vibration of the cation indicates that the carbonyl group of the cation is initially adsorbed in parallel to the Pt(111) surface at low coverage. The ν(NCN)_as_ vibration at 1542 cm^−1^ does not match the band position observed in the multilayers at 130 K (Figure S2) and 300 K (Figure 1), where we consistently observed the ν(NCN)_as_ vibration at 1572 cm^−1^. Furthermore, this band does not grow throughout the deposition. In contrast, the band at 794 cm^−1^, which we attributed to the δ(CH)_oop_ vibrations of the imidazole ring, grows consistently during IL deposition. Thus, we claim that at low coverage, the imidazole ring adsorbs in a largely parallel motif on the Pt(111) surface. However, due to the low but visible intensity of the (NCN)_as_ band, we assume that the ring is slightly inclined.

As coverage increases, the ν(CO) vibration at 1714 cm^−1^ begins to grow, while the ν(NCN)_as_ remains at 1542 cm^−1^ without further increase in intensity. Meanwhile, the δ(CH)_oop_ vibration continues to grow. This indicates that at higher coverage, the oxygen of the carbonyl group detaches from the surface, while the imidazole ring retains its parallel orientation on the Pt(111) surface.

At low coverage, the ν(SO₂)_as_ and ν(SNS)_as_ modes of the anion are not visible. However, with increasing coverage, both bands become prominent. Schuschke et al.^[46]^ found that the [NTf₂]^−^ anion initially adsorbs parallel to the surface with its oxygen atoms bound to the surface. This interpretation is supported by the low intensity of the ν(SO₂)_as_ vibration and the sharpness of the ν(CF₃)_as_ bands at low coverage. In line with these findings, we assume that the anion is oriented parallel to the surface and coordinates via the oxygen atoms of both SO₂ groups to the Pt(111) surface. The delayed appearance of the asymmetrical ν(SO₂)_as_ and ν(SNS)_as_ modes indicates that, at higher coverage, at least one SO₂ group detaches from the Pt(111) surface and is lifted up. This phenomenon was also observed in recent studies.[^23^, ^47^]

In addition to the IRAS experiments, we investigated a sub‐monolayer of [5‐oxo‐C_6_C_1_Im][NTf_2_] on Pt(111) by STM to obtain detailed information on the local adsorption and structure of the IL. We prepared a thin film of [5‐oxo‐C_6_C_1_Im][NTf_2_] on Pt(111) by PVD of the IL at 150 K under UHV conditions. Subsequently, we transferred the sample to the STM, which was cooled to temperatures below 200 K with liquid nitrogen. At this temperature, the IL is frozen, and surface diffusion can be inhibited. At the same time, the scanner unit was counter heated to RT.

**Figure 3:**
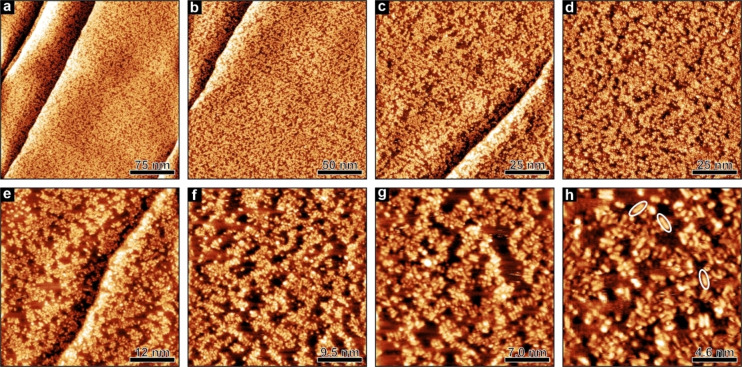
STM images of a [5‐oxo‐C_6_C_1_Im][NTf_2_] sub‐monolayer deposited on Pt(111) at 150 K at different scales: (a‐d) Overview images, (e‐h) molecular resolved images. Some cations are marked by white ovals. See Table S1 for detailed preparation and scanning parameters.

In Figure 3, STM images are shown of a Pt(111) surface covered with a sub‐monolayer of [5‐oxo‐C_6_C_1_Im][NTf_2_] (one monolayer (ML) represents a completely covered surface, i. e., a layer of adjacent anions and cations in direct contact with the surface). After we prepared the sample, the surface was partially covered with IL islands (Figure 3). The IL islands occur in different shapes and sizes. As we have already observed for [C_2_C_1_Im][OTf],^[22]^ there is no preferred adsorption site on Pt(111), e. g. at the step edges. Instead, the IL population is evenly distributed across the entire surface (Figure 3a–d). In comparison to different ILs on Au(111),[^35^, ^36^, ^50^, ^51^, ^52^, ^53^, ^54^] both [C_2_C_1_Im][OTf]^[22]^ and [5‐oxo‐C_6_C_1_Im][NTf_2_] form many small aggregates rather than a closed film on Pt(111).

**Figure 4:**
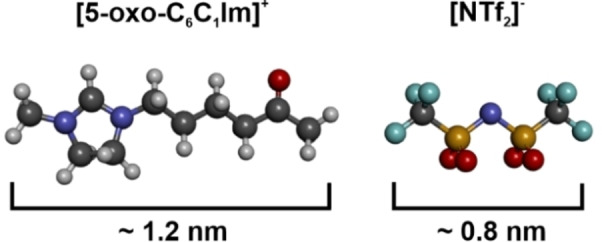
Ball model of [5‐oxo‐C_6_C_1_Im][NTf_2_] and approximate length of the two ions. The length of the cation [5‐oxo‐C_6_C_1_Im]^+^ is ~1.2 nm and that of the anion [NTf_2_]^−^ is ~0.8 nm.

At higher magnification, we are able to resolve the internal structure of the IL aggregates. We observe several different protrusions, which differ in brightness and shape. We assume that each feature represents an IL molecule, anion or cation, with a different adsorption motif. From literature[^47^, ^55^] we know that the [NTf_2_]^−^ anion can adsorb as cis or trans conformer. In addition, the different adsorption motifs differ by the number of oxygen molecules through which the [NTf_2_]^−^ binds to the surface. In total, six different adsorption motifs were found to be stable on Pt(111).^[47]^ Also, the [5‐oxo‐C_6_C_1_Im]^+^ cation can adsorb in different adsorption motifs (see IRAS and DFT). For example, the cation can lie flat on the surface, or at higher coverages, the side chain of the cation can stand up and point towards the vacuum. This phenomenon was also observed by Cremer et al. for [C_8_C_1_Im][NTf_2_] on Au(111).^[56]^ Some features appear more frequently, e. g. single or paired dots and elongated elliptic protrusions (Figure 3e–h). This indicates that some adsorption motifs are preferentially present at a specific coverage. In Figure 3h we marked some elongated features. We assign this feature to the [5‐oxo‐C_6_C_1_Im]^+^ cation with its chain lying flat on the surface. Not only the elongated shape of the feature indicates the flat lying cation, but also the length of the feature corresponds to the length of the cation (see Figures 4 and S3 for detailed analysis). In recent STM studies on imidazolium‐based cations on Au(111)[^53^, ^57^] and Ag(111),[^52^, ^54^] bright single dots have been observed. The authors attributed this feature to the cation with its chain pointing towards the vacuum.[^52^, ^53^, ^54^, ^57^] However, due to the large number of possible adsorption motifs on the surface, we refrain from further assignments.

**Figure 5:**
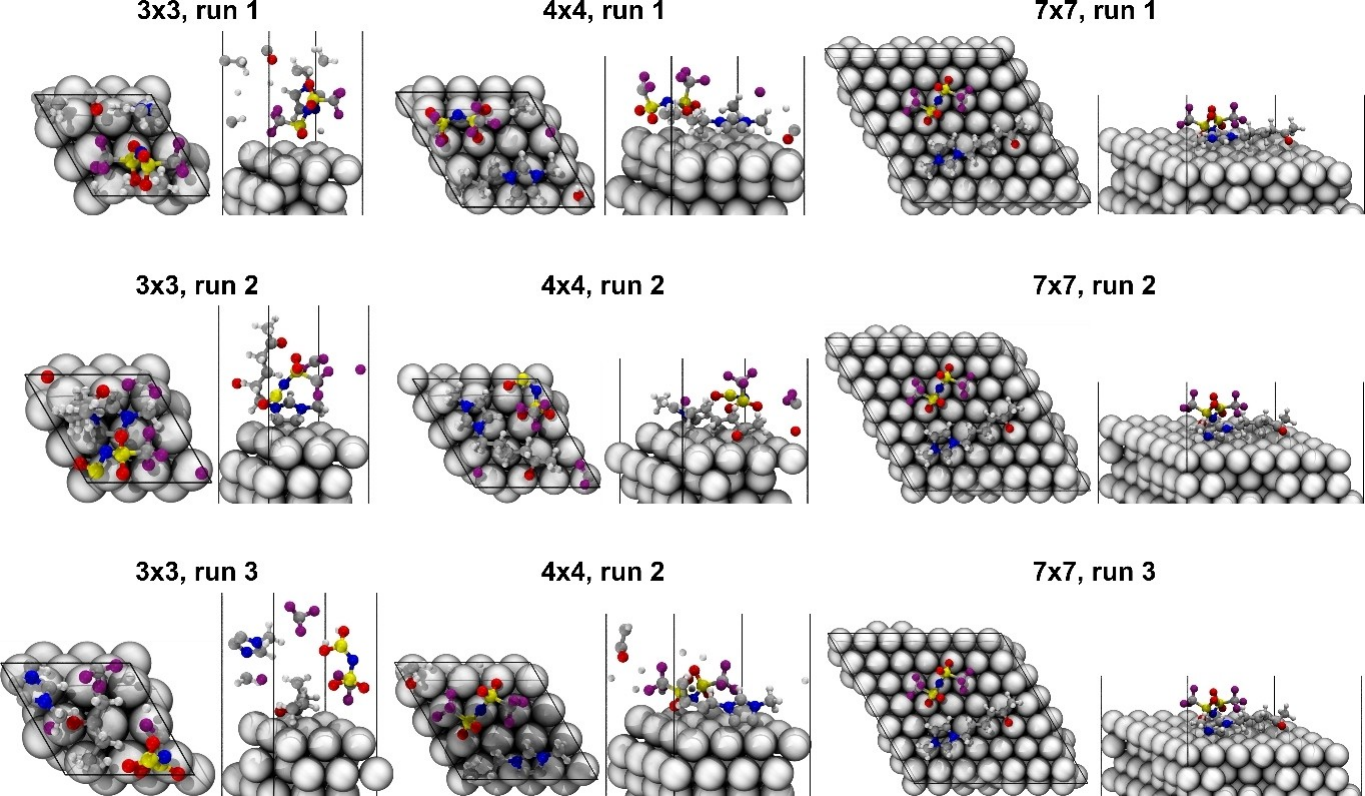
Optimized adsorption geometries of the [5‐oxo‐C_6_C_1_Im][NTf_2_] pairs optimized in surface slab unit cells of different sizes (3×3, 4×4, 7×7).

**DFT**. In order to identify preferred adsorption motifs of [5‐oxo‐C_6_C_1_Im][NTf_2_] on Pt(111), we performed simulations by DFT. Note that we focus our discussion on the [5‐oxo‐C_6_C_1_Im]^+^ cation, as a detailed study on the different adsorption motifs of the anion [NTf_2_]^−^ on Pt(111) can be found in literature.^[47]^ In this study, the [NTf_2_]^−^ anions were always placed in their trans‐conformation to limit the space of possible adsorption configurations. To simulate different coverages of [5‐oxo‐C_6_C_1_Im][NTf_2_] on Pt(111), surface slabs of different sizes (3×3, 4×4, 5×5, 6×6, and 7×7) were used. The IL molecules were placed on the surface in three different starting positions obtained from the lowest energy structures occurring during short MD runs at 700 K, and their geometries were optimized. The preferred adsorption motifs of the [5‐oxo‐C_6_C_1_Im][NTf_2_] pairs in the 3×3, 4×4 and 7×7 cells are shown in Figure 5, their energies are listed in Table T2 (for adsorption motifs and energies of all cells, see Figures S4 and S5).

Due to the small sizes of the unit cells, the IL ions cannot lay flat in the 3×3 and 4×4 cells. Instead, they are located either perpendicular to the surface or bended, such that the long cation is partly bound to the surface, with a part of its chain pointing upwards. Note that the ions appear as somewhat fragmented in the 3×3 and 4×4 cells, which is due to the inability of detecting chemical bonds for periodic systems within the vmd visualization program. In run 1, the cation is located perpendicular to the surface, whereas in run 2, the imidazolium ring is placed on the surface and in run 3, the carbonyl group is placed on the surface. Surprisingly, the adsorption of the imidazolium ring being partially chemisorbed on the surface (run 2) leads to a weaker total adsorption of the IL pair than run 1 and run 3. In the 4×4 unit cell, on the other hand, the adsorption is the strongest if the full cation lays flat and inclined on the surface. Here, only the ring is bound covalently to the surface. When the carbonyl is bound to the surface, the adsorption is weaker. From the 5×5 unit cell, both cation and anion lay flat on the surface. Comparing the adsorption energies of each unit cell size, we observe the flat adsorption in larger cells to be preferred (average E_ads_ (3×3, 4×4): 2.71 eV, average E_ads_ (5×5, 6×6, 7×7): 3.73 eV). The 7×7 unit cell has the highest average adsorption energy of 4.58 eV. The covalent adsorption of the carbonyl group to the Pt(111) surface (run 2 and run 3) shows higher adsorption energies. In both cases, the imidazolium ring is chemisorbed. Thus, we propose that the cation will be essentially fully chemisorbed if the surface coverage is much less than 1 ML. However, for higher surface coverage, we propose that the side chain of the cation straightens up, pointing towards the vacuum, in a similar way to run 2 in the 3×3 cells, starting from a flat‐lying cation with the imidazolium ring placed on the surface.

### Thermal Stability of [5‐oxo‐C6 C1Im][NTf2]

**Figure 6:**
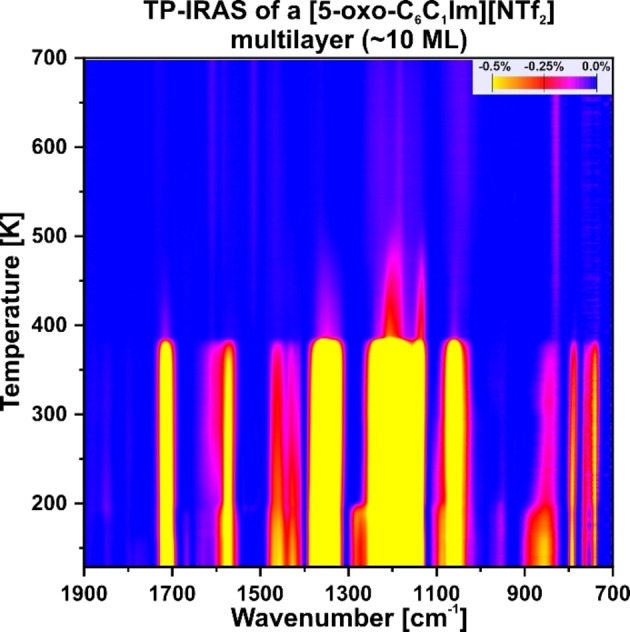
Temperature‐programmed IRAS experiment of [5‐oxo‐C_6_C_1_Im][NTf_2_] adsorbed on a clean Pt(111) surface. The thermal stability of the multilayer between 130 K and 700 K is shown.

**Multilayer**. In order to gain a more detailed understanding of the thermal stability and evolution of [5‐oxo‐C_6_C_1_Im][NTf_2_], we performed temperature programmed IRAS experiments. We applied a linear heating ramp of 2 K/min from 130 to 700 K while recording IRAS spectra every minute. In Figure 6 we show the absolute signals recorded between 130 and 700 K of a multilayer. The bands are at identical positions than in the previous experiments and can be assigned accordingly (see Figure 1 and Table 1). All signals remain stable up to 200 K. Afterwards, changes occur in the spectra. Massicot et al.^[21]^ studied the similar IL [C_1_C_1_Im][NTf₂] on Pt(111). They found that the IL initially adsorbs in a disordered structure. Upon heating to approximately 200 K, the IL reorganizes into a more ordered structure. Thus, we attribute the spectral changes at 200 K to a reordering effect within the multilayer. The multilayer desorbs in a stepwise manner at 390 K. After this temperature, a monolayer, which is more strongly bound to the surface, remains. Note that the absence of the ν(NCN)_as_ and δ(CH) vibration modes in this temperature region is most likely attributed to their intrinsically much lower intensity as compared to the ν(CO) vibration and the anion bands. As the temperature increases, the intensity of the monolayer bands decreases steadily. Although the ν(CO) vibration disappears at 460 K, some anion bands remain visible up to 530 K. As desorption occurs in form of ion pairs, we attribute this to the decomposition of the IL, with the cation fragment desorbing earlier than the anion fragment.

**Figure 7:**
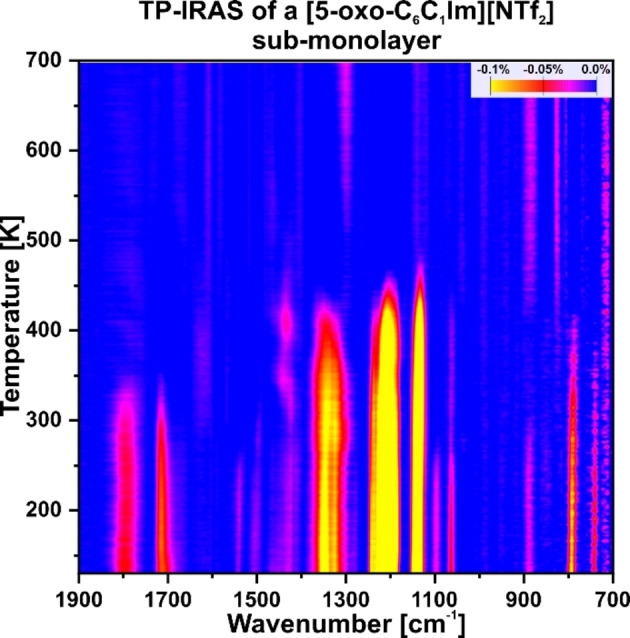
Temperature‐programmed IRAS experiment of [5‐oxo‐C_6_C_1_Im][NTf_2_] adsorbed on a clean Pt(111) surface, displaying the thermal stability of the sub‐monolayer between 130 K and 700 K.

**(Sub−)monolayer**. Additionally, we investigated the thermal stability and evolution of a [5‐oxo‐C_6_C_1_Im][NTf_2_] sub‐monolayer, which is in direct contact with the surface, by TP‐IRAS. IRAS spectra were recorded every minute while applying a linear heating ramp of 2 K/min between 130 and 700 K (Figure 7).

**Figure 8:**
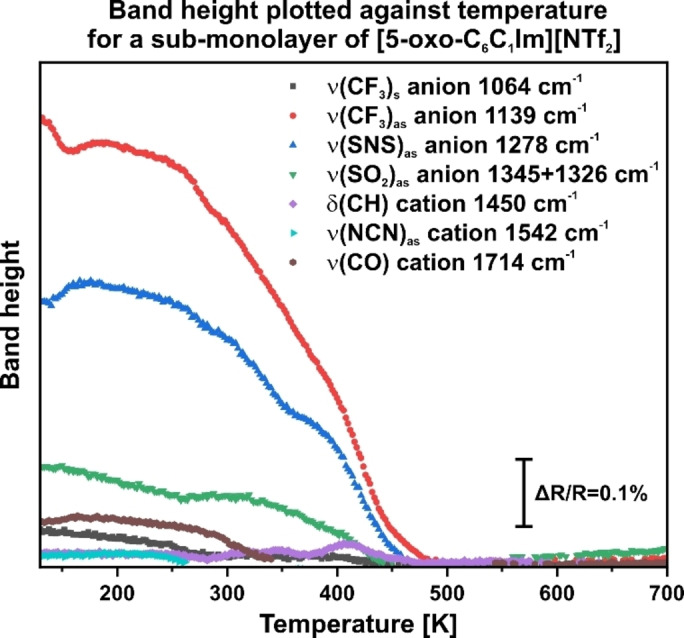
Sub‐monolayer of [5‐oxo‐C_6_C_1_Im][NTf_2_] adsorbed on a clean Pt(111) surface. The band height is displayed as a function of the temperature.

At 130 K, we observe bands similar to those during the deposition experiment (see Figure S2). The cation bands appear at 1714, 1542, 1426, and 794 cm^−1^, while the anion bands are at 1348, 1326, 1218, 1139, and 1064 cm^−1^. The bands can be assigned accordingly to the previous experiments (see Figure 1 and Table 1). Figure 8 illustrates the height of bands for both the cation and anion relative to the temperature. Both, Figures 7 and 8, demonstrate that the bands remain stable between 130 and 250 K, followed by a gradual decrease. The cation bands disappear between 250 and 350 K. However, at 350 and 400 K, new δ(CH) signals emerge at 1450 cm^−1^. The anion bands also decrease until approximately 500 K, at which point no IL signals remain. Recent studies indicate that other imidazole‐based ILs begin to decompose at around 250–300 K.[^19^, ^21^, ^22^] Thus, we propose that [5‐oxo‐C_6_C_1_Im][NTf_2_] starts to decompose and partially desorbs at 250 K. We assign the species forming at 1450 cm^−1^ at 350 and 400 K to decomposition products.

**Figure 9:**
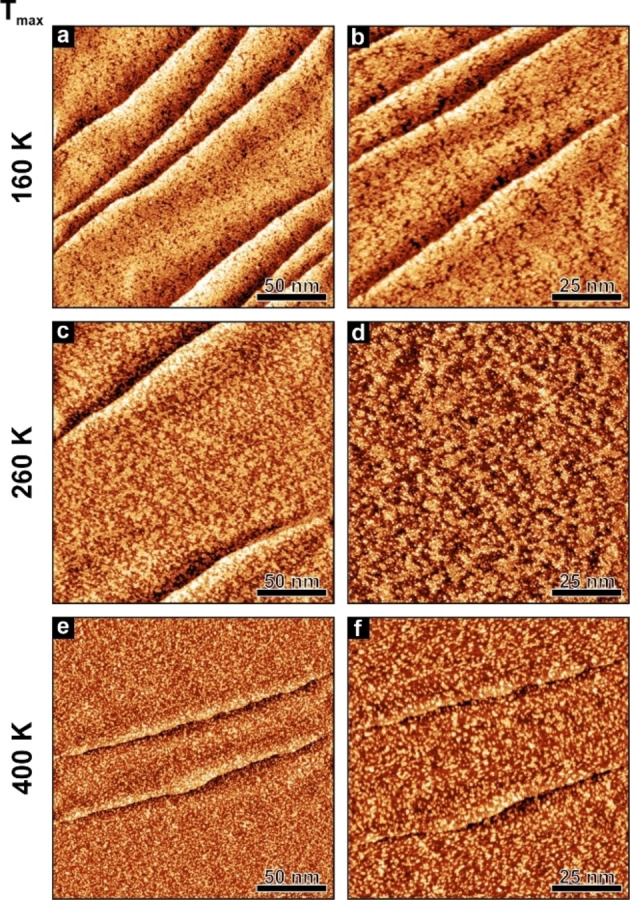
STM images of a [5‐oxo‐C_6_C_1_Im][NTf_2_] sub‐monolayer on Pt(111); (a‐b) measured after deposition at 160 K, (c‐d) after annealing to 260 K, and (e‐f) after annealing to 400 K. All STM images were measured at 100 K. See Table S1 for detailed preparation and scanning parameters.

In addition, we performed temperature‐dependent STM experiments by heating the sample to a desired temperature, holding it at that temperature, and then cooling it back to the measurement temperature. In Figure 9, we present the thermal evolution of a [5‐oxo‐C_6_C_1_Im][NTf_2_] sub‐monolayer at different temperatures. The images are recorded immediately after deposition of [5‐oxo‐C_6_C_1_Im][NTf_2_] at 160 K and after heating to 260 and 400 K, respectively. For each temperature, two representative overview images with different magnification are shown.

Directly after preparation at 160 K, the Pt(111) surface is covered with islands of [5‐oxo‐C_6_C_1_Im][NTf_2_]. As already discussed before, the IL does not form a closed film but many small aggregates, which are evenly distributed across the surface. The coverage is close to 1 ML (Figure 9a and b). After annealing to 260 K, we observe fewer IL islands (Figure 9c and d). Between the islands, the bare Pt(111) surface is visible. In addition, less bright protrusions emerge between the IL islands in the background. Based on our findings from TP‐IRAS and STM experiments, we assume that at this temperature, the IL starts to decompose and, thus, the intact IL and decomposition products coexist on the surface. Furthermore, IRAS, and STM indicate partial desorption of the IL. Further heating to 400 K leads to even larger changes (Figure 9e and f). The coverage decreases dramatically, with only a few small IL islands remaining on the surface. Additionally, many protrusions appear as dots, as we already found for [C_2_C_1_Im][OTf] on Pt(111)^[22]^ at elevated temperatures. We assume that the IL decomposes further at 400 K. Therefore, mainly the decomposition products are adsorbed on the surface. Note that we performed additional temperature dependent experiments where we annealed the sample at 150 K, 200 K, 260 K and 300 K (Figure S6). We observe comparable results in this series of measurements (see Figure S6). A similar behavior was found for [C_2_C_1_Im][OTf] on Pt(111),^[22]^ [C_1_C_1_Im][NTf_2_] on Cu(111),^[19]^ and [C_1_C_1_Im][NTf_2_] on Pt(111)^[21]^ already. The authors proposed that a surface reaction results in chemical decomposition. Depending on the IL, the onset of decomposition is between 250 and 300 K.[^19^, ^21^, ^22^]

## Summary and Conclusions

In summary, we studied the growth and adsorption of a thin film of [5‐oxo‐C_6_C_1_Im][NTf_2_] on Pt(111) at low temperature. In addition, we investigated the thermal stability of [5‐oxo‐C_6_C_1_Im][NTf_2_] by temperature‐dependent experiments. We summarize our main results as follows:

### Adsorption

- At low coverage and temperatures below 200 K, PVD of [5‐oxo‐C_6_C_1_Im][NTf_2_] on Pt(111) results in the growth of many small IL aggregates rather than a closed film. The islands are evenly distributed across the Pt terraces.
- The [5‐oxo‐C_6_C_1_Im]^+^ cation adsorbs with the imidazole ring lying flat on the surface. The carbonyl group is oriented parallel to the surface. With increasing coverage, the carbonyl group disconnects from the surface and lifts up while the imidazole ring remains on the surface.
- The [NTf_2_]^−^ anion adsorbs parallel to the surface via the oxygen atoms of the SO_2_ groups. With increasing coverage, at least one of the SO_2_ groups completely detaches from the surface, pointing towards the vacuum.
- In the multilayer, the IL molecules are physisorbed in a random orientation.

### Thermal Stability

- At coverages below 1 ML, decomposition and partial desorption of [5 oxo‐C_6_C_1_Im][NTf_2_] starts at around 260 K. Between 350 and 400 K, further decomposition and desorption occurs. Above 500 K, only residues are left on the surface.

- The [5‐oxo‐C_6_C_1_Im][NTf_2_] multilayer undergoes a reorientation to a more ordered structure at 200 K and desorbs intact at 390 K. Above this temperature, decomposition takes place, with the cation fragment desorbing earlier than the anion fragment.

## Conflict of Interests

The authors declare no conflict of interest.

1

## Supporting information

As a service to our authors and readers, this journal provides supporting information supplied by the authors. Such materials are peer reviewed and may be re‐organized for online delivery, but are not copy‐edited or typeset. Technical support issues arising from supporting information (other than missing files) should be addressed to the authors.

Supporting Information

## Data Availability

The data that support the findings of this study are presented in the Manuscript. Source data are provided at Zenodo: https://doi.org/10.5281/zenodo.13959623
