# Supplementary material for: Adsorption and Thermal Evolution of the Carbonyl‐functionalized Ionic Liquid [5‐oxo‐C6C1Im][NTf2] on Pt(111): A Combined IRAS, STM, and DFT Study
Source: Chemistry. 2024 Dec 10;31(8):e202403900. doi: 10.1002/chem.202403900 (PMC11803367; doi:10.1002/chem.202403900)
Supplement: Supplementary file 1 — Supporting Information [file CHEM-31-e202403900-s001.pdf]

Chemistry–A European Journal

Supporting Information

Adsorption and Thermal Evolution of the Carbonyl-functionalized Ionic Liquid [5-oxo-C_6C_1Im][NTf_2] on Pt(111): A Combined IRAS, STM, and DFT Study

Hanna Bühlmeyer, Lukas Knörr, Julien Steffen, Roman Eschenbacher, Jonas Hauner, Andreas Görling, and Jörg Libuda*

Supporting Information

Adsorption and Thermal Evolution of the Carbonyl-Functionalized Ionic Liquid [5-oxo-C_6C_1Im][NTf_2] on Pt(111): A Combined IRAS, STM, and DFT Study

Hanna Bühlmeyer[1§], Lukas Knörr[1§], Julien Steffen[2], Roman Eschenbacher[1], Jonas Hauner[1], Andreas Görling[2], Jörg Libuda[1*]

[1] *Interface Research and Catalysis, ECRC, Friedrich-Alexander-Universität Erlangen-Nürnberg, Egerlandstraße 3, 91058 Erlangen, Germany*

[2] *Chair of Theoretical Chemistry, Friedrich-Alexander-Universität Erlangen-Nürnberg, Egerlandstraße 3, 91058 Erlangen, Germany*

§these authors contributed equally

*corresponding author: Jörg Libuda, joerg.libuda@fau.de

Table S1: Detailed preparation and scanning parameter of all STM images.

Figure	T_{max} sample	T_{prep} sample	T_{STM}	U_b	I_T
3a	200 K	150 K	100 K	1.5 V	220 pA
3b	200 K	150 K	100 K	1.5 V	230 pA
3c	200 K	150 K	200 K	1.5 V	220 pA
3d	200 K	150 K	100K	1.5 V	220 pA
3e	200 K	150 K	100 K	1.5 V	220 pA
3f	200 K	150 K	100 K	1.5 V	210 pA
3g	200 K	150K	100 K	1.5 V	240 pA
3h	200 K	150 K	100 K	1.5 V	220 pA
9a	160 K	160 K	100 K	1.6 V	220 pA
9b	160 K	160 K	100 K	1.6 V	220 pA
9c	260 K	160 K	100 K	1.5 V	220 pA
9d	260 K	160 K	100 K	1.5 V	220 pA
9e	400 K	160 K	100 K	1.5 V	310 pA
9f	400 K	160 K	100 K	1.5 V	280 pA
S5a	150 K	150 K	100 K	1.5 V	220 pA
S5b	150 K	150 K	100 K	1.5 V	230 pA
S5c	200 K	150 K	200 K	1.5 V	220 pA
S5d	200 K	150 K	200 K	1.5 V	220 pA
S5e	260 K	150 K	100 K	1.2 V	770 pA
S5f	260 K	150 K	100 K	1.2 V	720 pA
S5g	300 K	150 K	100 K	1.5 V	250 pA
S5h	300 K	150 K	100 K	1.5 V	260 pA

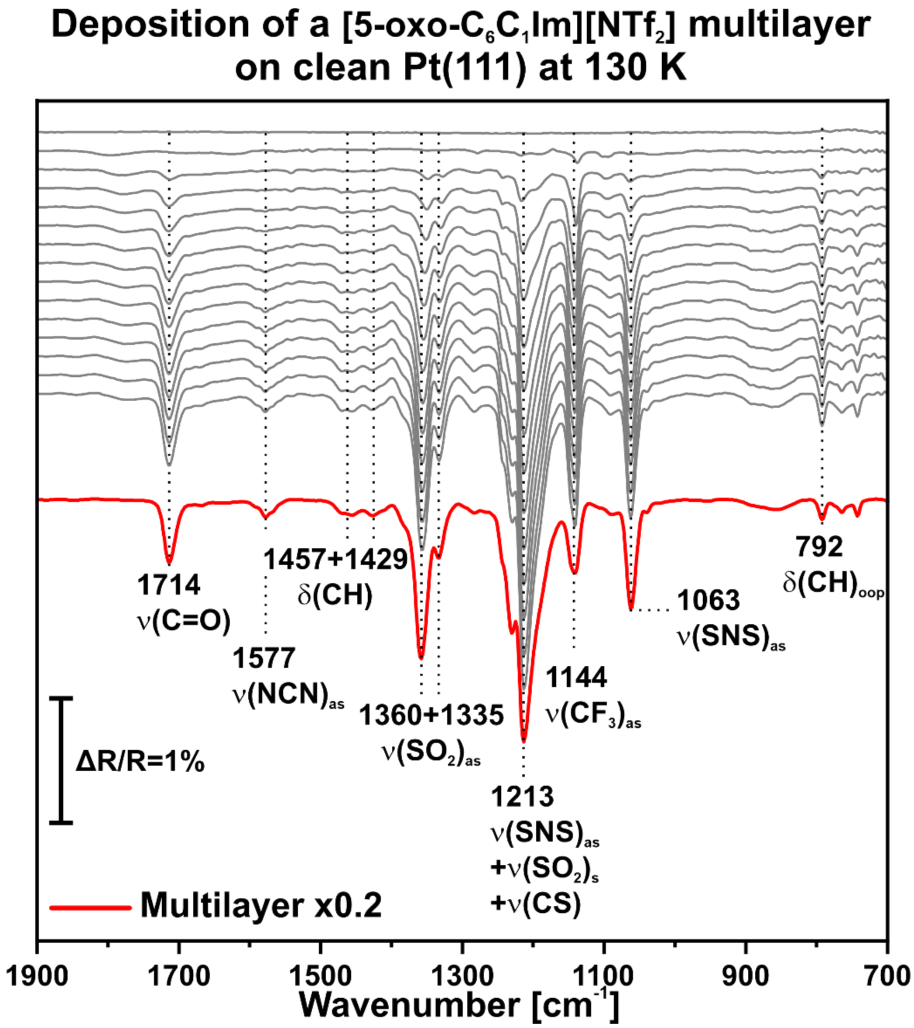

Figure S2: Deposition of [5-oxo-C_6C_1Im][NTf_2] on a clean Pt(111) surface at 130 K recorded by time-resolved IRAS. The spectrum at multilayer coverage is highlighted in red and scaled by 0.2.

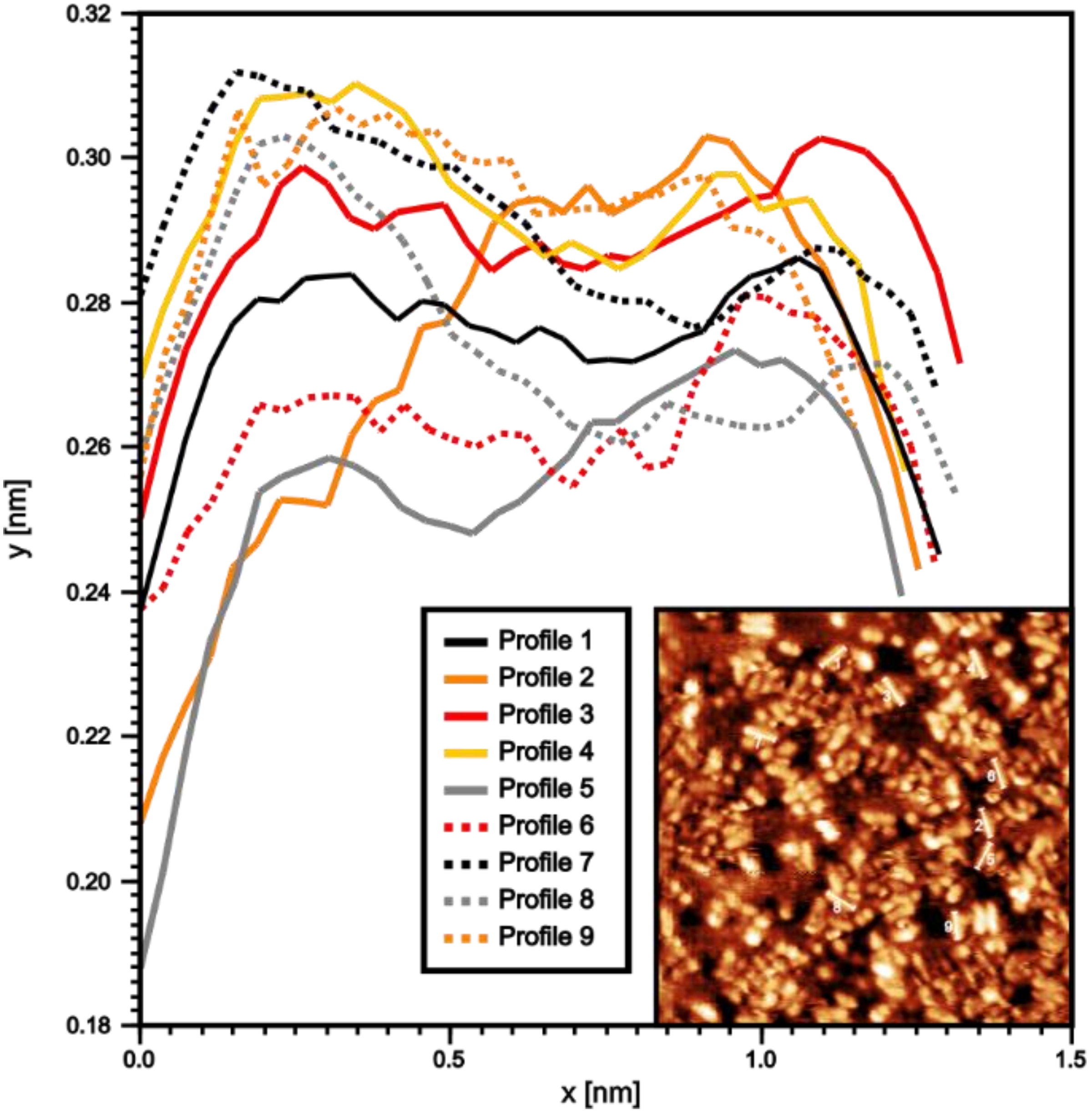

Figure S3: Line profiles of various selected elongated features in the STM image (the STM image is similar to the one shown in **Figure 3h)**. The line profiles are labelled with the corresponding number in the STM image. The length of the features is ~ 1.2 nm.

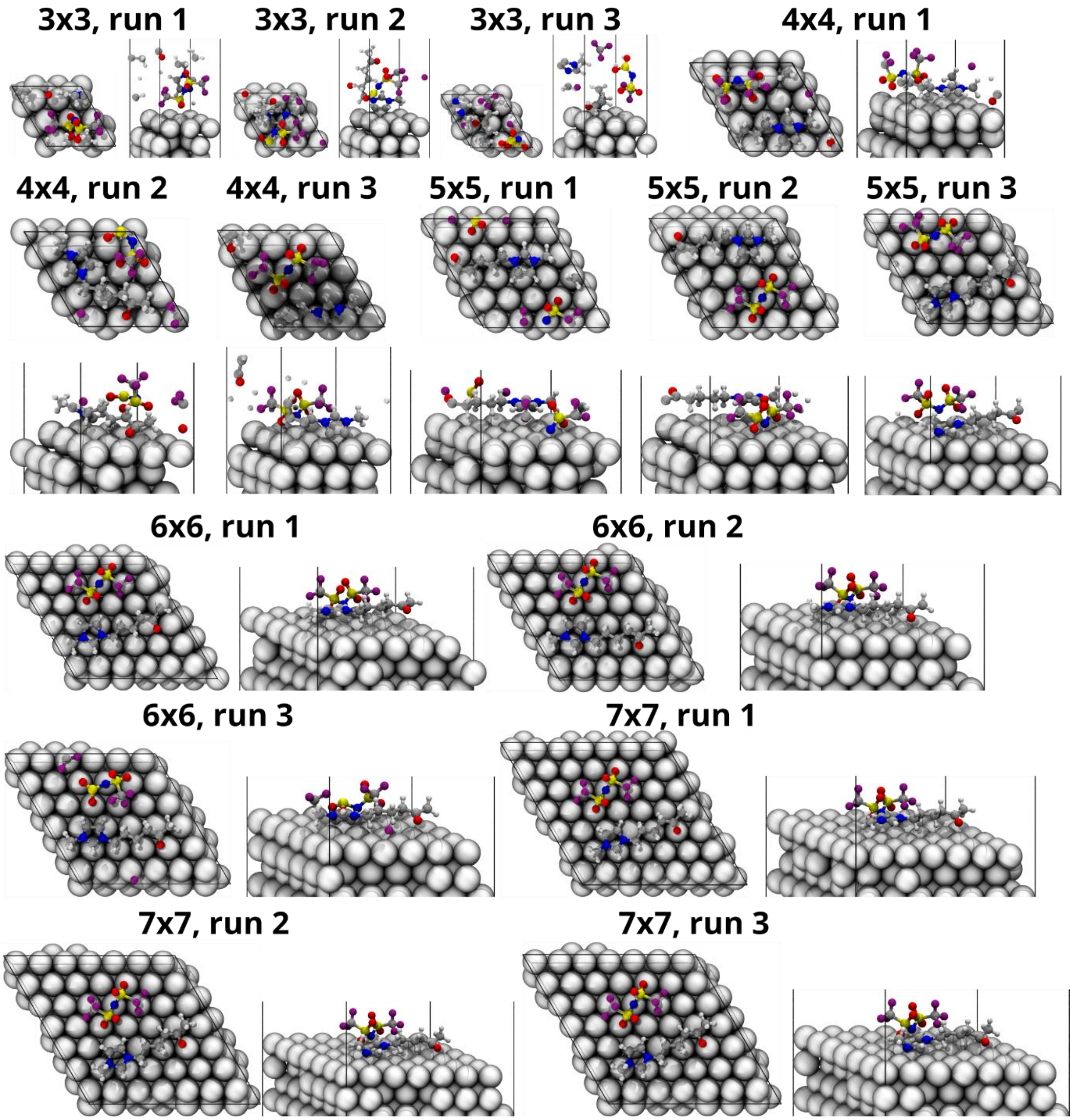

Figure S4: Optimized adsorption geometries of the [5-oxo-C_6C_1Im][NTf_2] pairs optimized in surface slab unit cells of all different sizes (3x3, 4x4, 5x5, 6x6, 7x7).

Table S5: Total energies and adsorption energies of [5-oxo-C_6C_1Im][NTf_2].

Species	Energy [eV]	Adsorption energy [eV]
3x3 (run 1)	-553.161513	2.179461
3x3 (run 2)	-552.790481	1.808429
3x3 (run 3)	-553.417679	2.435627
4x4 (run 1)	-779.820547	3.221349
4x4 (run 2)	-779.046382	2.447184
4x4 (run 3)	-780.768040	4.168842
5x5 (run 1)	-1070.587312	3.989546
5x5 (run 2)	-1070.484879	3.887113
5x5 (run 3)	-1070.804839	4.207073
6x6 (run 1)	-1424.376401	3.717901
6x6 (run 2)	-1424.165626	3.507126
6x6 (run 3)	-1424.676039	1.017539
7x7 (run 1)	-1843.480765	4.262920
7x7 (run 2)	-1843.964671	4.746826
7x7 (run 3)	-1843.925374	4.707529

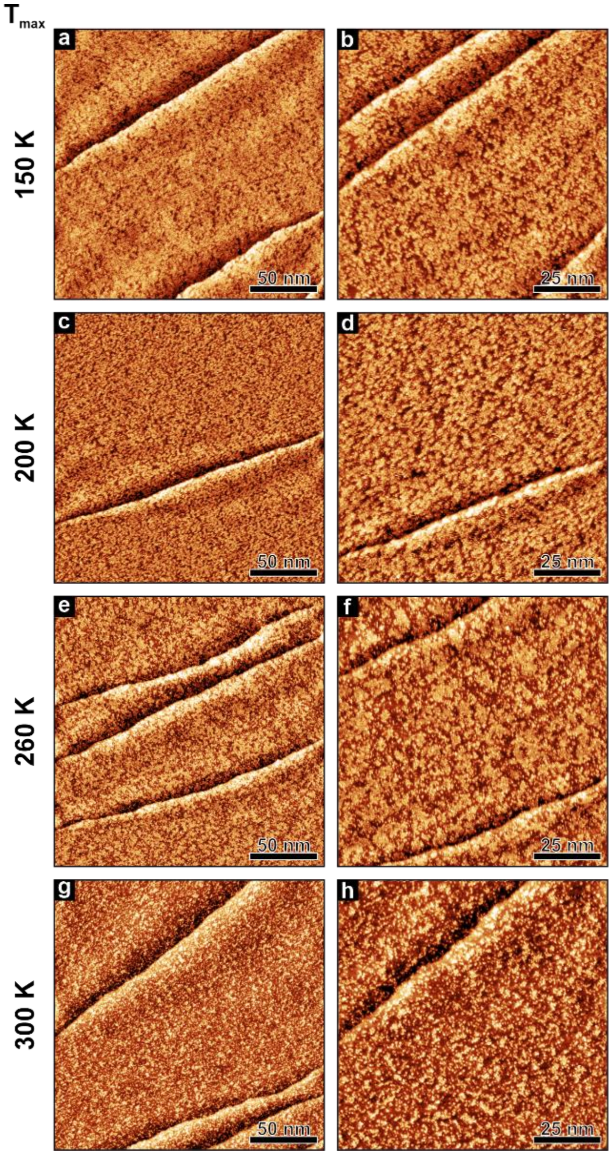

Figure S6: STM images of a [5-oxo-C_6C_1Im][NTf_2] sub-monolayer on Pt(111); (a-b) measured after deposition at 150 K, (c-d) after annealing to 200 K, and (e-f) after annealing to 260 K, (g-h) after annealing at 300 K for 5 days. All STM images were measured at 100 K. See **Table T1** for detailed preparation and scanning parameters.
